# Ways to improve the negative impact of proton pump inhibitors on biopsy-based test accuracy for *Helicobacter pylori* detection

**DOI:** 10.1007/s12664-025-01841-0

**Published:** 2025-08-05

**Authors:** Peter Malfertheiner, Christian Schulz

**Affiliations:** 1 Medical Department II, University Hospital, Ludwig-Maximilians-Universität, Munich, Germany; 2 DZIF Deutsches Zentrum Für Infektionsforschung, Partner Site Munich, Munich, Germany

The administration of proton pump inhibitors (PPIs) significantly interferes with the accuracy of all diagnostic tests, except serology, used to detect *Helicobacter pylori* (*H. pylori*) and false-negative results due to PPI administration have significant implications for the management of *H. pylori* infection [1].

Clinical guidelines therefore recommend discontinuing PPI therapy for two weeks before performing urea breath test (UBT), stool antigen test (SAT) and gastric biopsy-based tests (GBT) [2, 3]. However, in clinical practice, discontinuation of PPI is often not feasible.

The authors, Yadav and colleagues, of the study published in this issue of the *Indian Journal of Gastroenterology* deserve credit for addressing the important clinical question of whether, with continued use of PPIs within two weeks before upper gastrointestinal endoscopy (UGE), a way can be found to improve the diagnostic sensitivity of GBT for detecting *H. pylori* [4]. They explored and compared GBT for *H. pylori* detection in 164 patients with dyspeptic symptoms undergoing UGE. Polymerase chain reaction (PCR)  was confirmed as the most accurate diagnostic method and there was no difference in diagnostic sensitivity when patients on PPI were compared to a separate sub-group of 50 patients off PPI. This is an expected finding, as only tiny bacterial DNA amounts of not necessarily vital organisms are required for *H. pylori* detection by PCR. In the presence of strong acid suppression with PPI, bacterial density is dramatically reduced and diagnostic sensitivity is low for standard routine tests performed on gastric biopsies under such conditions. Although PCR is advisable as the method of choice in patients on PPI, limited availability and costs, according to the authors, restrict its widespread use; and this is certainly an important consideration and not limited to clinical practice in India. The authors therefore sought to improve the conventional testing procedure with rapid urease testing (RUT) by combining two biopsies from two stomach regions into one test sample. When compared with PCR as the gold standard, which is the most suitable comparator, the modified RUT on two gastric biopsies combined from two sites, antrum and corpus, was 43.7% and a similar performance of 40% was shown for histopathology (HPT) according to the updated Sydney Classification. A further comparative analysis considered the *H. pylori* status as positive if two of three tests among PCR, dual-site RUT and HPT were positive. The definition of a diagnostic gold standard that relies on the same tests that are investigated for their accuracy appears not appropriate, but what may be of potential concern is the requirement of two positive tests to define a patient as *H. pylori* positive. What about patients with one test only positive? We assume that the authors of the article would agree with the recommendation to initiate eradication therapy even if only one of the test results is positive.

Overall, the diagnostic sensitivity of the two conventional methods, the double-site RUT and HPT, remains low. The authors agree in their conclusions that the diagnostic improvement would not justify to remove the current guideline recommendations that advises to stop PPI intake two weeks prior to performing any conventional diagnostic procedure. PCR is an alternative to this, as the sensitivity in the detection of *H. pylori* is not influenced by ongoing PPI, but has the previously mentioned restrictions.

At this point, the question arises whether the study concept of the authors to rely on dual-site biopsies rather than on a single biopsy-based RUT in patients on PPI offers room for improvement. For exploring this further, we need to consider the changes in gastric physiology and direct effects on *H. pylori* induced by PPI. In brief, by increasing the gastric pH, PPI profoundly change the intra-gastric distribution of *H. pylori*, with a critical reduction or even complete disappearance in the antrum and a shift of the bacteria to a more proximal, oxyntic mucosa containing gastric corpus and fundus region [5]. This suggests that, for improving the detection rate of *H. pylori* for RUT from biopsies of two sites, it would seem to be more appropriate to take biopsies from the corpus and fundus mucosa rather than a biopsy from the antrum. A comparison of the individual *H. pylori* detection sensitivity between the two sites, antrum and corpus, for further confirmation of this knowledge was not performed in the study by Yadav and colleagues.

PPI have also a direct effect on *H. pylori* by inhibiting urease activity and this effect is most relevant concerning the sensitivity of the UBT. Urease inhibition by PPI is dose dependent and a negative influence applies to RUT sensitivity as well. Such effect, however, was not apparent in the study by Yadav and colleagues, as the sensitivity of RUT from two sites corresponds to the histopathological detection rate of *H. pylori* which is not at all related to urease activity. In the specific case, the finding is likely due to the low or standard dose of PPI used by the patients in the study.

Taken the findings from the study presented in this issue of the *Indian Journal of Gastroenterology* and current knowledge on the effect of PPI on the accuracy of testing influenced by changes in the “intra-gastric behavior” of *H. pylori* by PPI, the rationale of testing patients on PPI presenting to UGE would encourage taking biopsies for RUT from the fundus-corpus mucosa, combined with the histological examination. A confirmatory study to prove the concept is needed. A further aspect to be taken into account may be the reading time of the RUT, which should take more than the usual 30 minutes and be extended to several, up to eight  hours (according to our experience) as the low density of bacterial colonization requires more time for the urease enzyme reaction.

## Data Availability

There are no data associated with this editorial.

## Declarations

### Disclaimer

The authors are solely responsible for the data and the content of the paper. In no way, the Honorary Editor-in-Chief, Editorial Board Members, the Indian Society of Gastroenterology, printer/publishers, or are responsible for the results/findings and content of this article.
